# Nephrolithiasis predicts ischemic stroke: A longitudinal follow-up study using a national sample cohort

**DOI:** 10.7150/ijms.34417

**Published:** 2019-07-21

**Authors:** So Young Kim, Chang Myeon Song, Woojin Bang, Jae-Sung Lim, Bumjung Park, Hyo Geun Choi

**Affiliations:** 1 Department of Otorhinolaryngology-Head & Neck Surgery, CHA Bundang Medical Center, CHA University, Seongnam, Korea; 2 Department of Otorhinolaryngology-Head & Neck Surgery, Hanyang University College of Medicine, Seoul, Korea; 3 Department of Urology, Hallym University College of Medicine, Anyang, Korea; 4 Department of Neurology, Hallym University Sacred Heart Hospital, Anyang, Korea; 5 Department of Otorhinolaryngology-Head & Neck Surgery, Hallym University College of Medicine, Anyang, Korea; 6 Hallym Data Science Laboratory, Hallym University College of Medicine, Anyang, Republic of Korea

**Keywords:** nephrolithiasis, kidney calculi, stroke, infarct, cohort studies, nested case-control studies.

## Abstract

This study sought to evaluate associations between nephrolithiasis and hemorrhagic and ischemic stroke using a national sample cohort from Korea. Data from 2002 to 2013 were collected for individuals ≥ 20 years of age in the Korean National Health Insurance Service (NHIS)-National Sample Cohort. We extracted nephrolithiasis patients (n = 22,636) and 1:4 matched controls (n = 90,544) and analyzed the occurrence of stroke. Matching was performed based on age, sex, income, region of residence, hypertension, diabetes mellitus, and dyslipidemia history. Crude and adjusted hazard ratios (HRs) were calculated using Cox proportional hazard models, and 95% confidence intervals (CIs) were determined. We performed subgroup analyses according to age, sex, and follow-up period. The adjusted HR of ischemic stroke was 1.13 (95% CI = 1.06-1.21) in the nephrolithiasis group (P < 0.001). The relationship between nephrolithiasis and ischemic stroke remained present for the young women and middle-aged men as well as during a follow-up period of ≤ 1 year. The HR for hemorrhagic stroke did not reach statistical significance. The risk of ischemic stroke was higher in the nephrolithiasis patients.

## Introduction

Nephrolithiasis refers to a stone in a kidney or lower in the urinary tract. The prevalences of this condition have been reported to be 10.6% in men and 7.1% in women in the USA 1 and 5.0% in Korea.^2^ The annual incidence was estimated to be 457 per 100,000 in Koreans.^3^ At present, the exact pathophysiology of renal stone formation remains unclear. Various risk factors have been proposed, such as chronic kidney disease; poor hydration; abnormal calcium metabolism, including hyperparathyroidism; increasing age; obesity; diabetes mellitus; warm climate; and high animal protein intake.^4^-^7^

Associations between nephrolithiasis and hypertension, dyslipidemia, diabetes mellitus, myocardial infarction, and stroke have previously been reported.^8^-^11^ Obesity, insulin resistance,^12^ hypercalciuria and vascular calcification have been suggested as possible pathophysiologies of nephrolithiasis.^13^ Prior results have been inconsistent with respect to a potential association between nephrolithiasis and stroke. Certain studies failed to find a relationship between nephrolithiasis and stroke after adjusting for possible confounders,^14^,^15^ whereas other investigations indicate the existence of this relationship.^11^,^16^ Two recent meta-analyses reported a positive association between nephrolithiasis and stroke.^17^,^18^ However, few studies have divided stroke into hemorrhagic and ischemic stroke.

The purpose of this study was to evaluate associations between nephrolithiasis and stroke using a national sample cohort of the Korean population. We extracted nephrolithiasis patients and 1:4 matched controls and analyzed the occurrence of stroke. In this study, we divided stroke into hemorrhagic and ischemic stroke. In addition, we performed analyses based on follow-up periods.

## Materials and Methods

### Study Population and Data Collection

The ethics committee of Hallym University (2017-I102) approved the use of the study data. The requirement for written informed consent was waived by the university's institutional review board. All methods were performed in accordance with the guidelines and regulations of the ethic committee of Hallym University.

This national cohort study relies on data from the Korean Health Insurance Review and Assessment Service-National Sample Cohort (HIRA-NSC). The detailed description of this data was described in our previous studies 19,20.

### Participant Selection

Among 1,125,691 patients with 114,369,638 medical claim codes, we included individuals who were diagnosed with nephrolithiasis (ICD-10: N20, calculus of kidney and ureter). Among these individuals, we selected patients who were treated ≥ 2 times (n = 24,123).

Histories of admission for hemorrhagic stroke (I60: subarachnoid hemorrhage, I61: intracerebral hemorrhage, and I62: other non-traumatic intracranial hemorrhage) and ischemic stroke (I63: cerebral infarction) were identified using ICD-10 codes. We selected participants who were treated for stroke ≥ 1 time. These methods were used in other studies that evaluated the incidence of stroke in Korea.^21^,^22^

**Figure 1:**
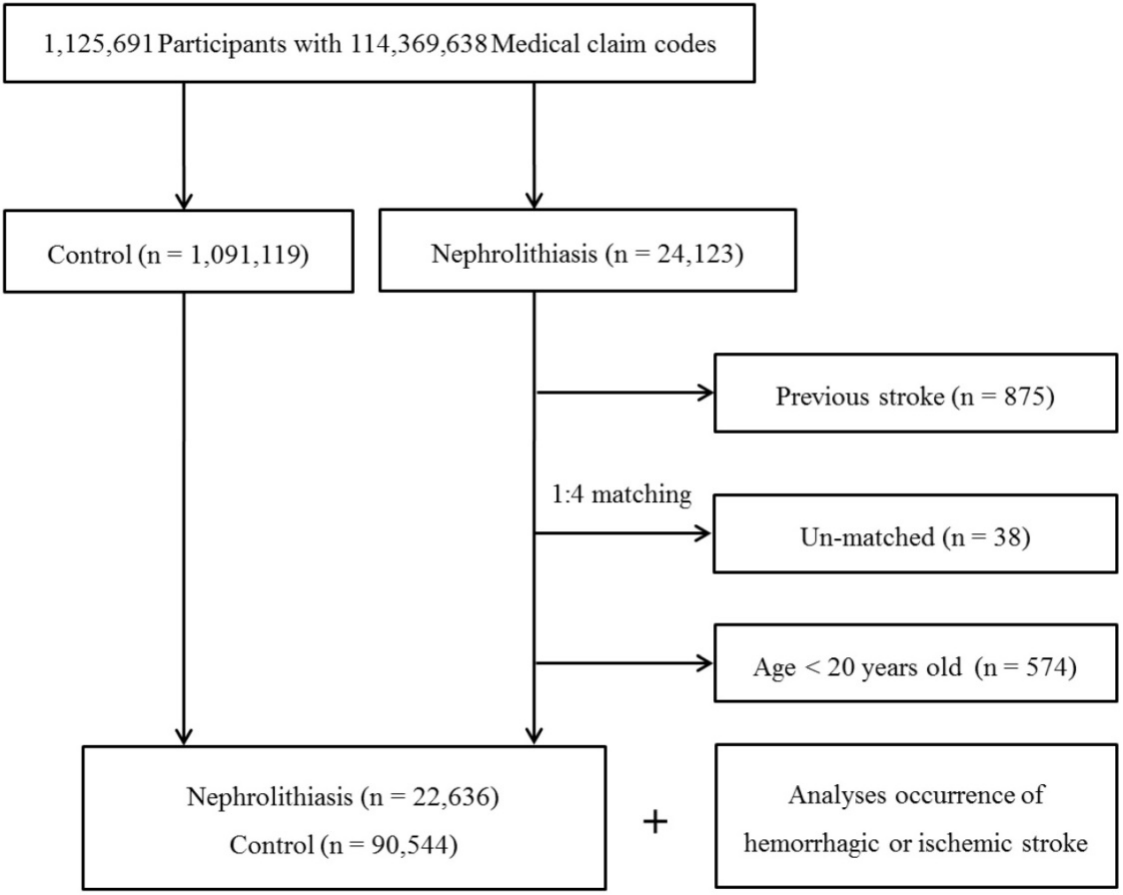
A schematic illustration of the participant selection process used in the present study. Out of a total of 1,125,691 participants, 22,636 nephrolithiasis patients were matched with 90,544 control participants based on age, group, sex, income group, region of residence, and prior medical history.

The nephrolithiasis subjects were matched 1:4 with subjects in the cohort who were never diagnosed with nephrolithiasis from 2002 to 2013 (the control group). The control group was selected from the mother population (n = 1,091,119). Matching was performed based on age, group, sex, income group, region of residence, and prior medical history (hypertension, diabetes, and dyslipidemia). To prevent selection bias when choosing the matched participants, the potential control group subjects were sorted using a random number order and were then selected from top to bottom. It was assumed that each nephrolithiasis patient and the matching control participants were receiving any needed medical treatment during concurrent time periods (based on the relevant index date). Therefore, the subjects in the control group who died before the index date were excluded. Because of index date matching, the follow up periods were almost same in both nephrolithiasis participants (72.1 months, Standard deviation [SD] = 41.4) and control participants (72.1 months, SD = 41.4). In both the nephrolithiasis and control groups, the participants with a history of hemorrhagic or ischemic stroke prior to the index date were excluded. In the nephrolithiasis group, 875 participants were excluded. The nephrolithiasis patients for whom we could not identify enough matching participants were excluded (n = 38). We also excluded the individuals under 20 years of age (n = 574). Finally, 1:4 matching resulted in the inclusion of 22,636 nephrolithiasis patients and 90,544 control participants (Fig. 1). However, the study subjects were not matched with respect to ischemic heart disease or history of depression because strict matching based on these characteristics increased the drop-out rate of the subjects due to a lack of control participants.

### Variables

The following age groups were defined using 5-year intervals: 20-24, 25-29, 30-34…, and 85+ years. A total of 14 age groups were designated. The income groups were initially divided into 41 classes (one health aid class, 20 self-employment health insurance classes, and 20 employment health insurance classes). These groups were re-categorized into 11 classes (class 1 [lowest income]-class 11 [highest income]). Region of residence was divided into 16 areas based on administrative district. These regions were regrouped into urban (Seoul, Busan, Daegu, Incheon, Gwangju, Daejeon, and Ulsan) and rural (Gyeonggi, Gangwon, Chungcheongbuk, Chungcheongnam, Jeollabuk, Jeollanam, Gyeongsangbuk, Gyeongsangnam, and Jeju) areas.

The participants' prior medical histories were evaluated using ICD-10 codes. To ensure the accuracy of diagnoses, hypertension (I10 and I15), diabetes (E10-E14), and dyslipidemia (E78) were regarded as present if a participant was treated ≥ 2 times. Ischemic heart disease (I24 and I25) was regarded as present if a participant was treated ≥ 1 time. Depression was defined based on the ICD-10 codes from F31 (bipolar affective disorder) to F39 (unspecified mood disorder) recorded by a psychiatrist.

### Statistical Analyses

Chi-square tests were used to compare the rates of the general characteristics between the nephrolithiasis and control groups.

Cox proportional hazard models were used to analyze hazard ratios (HR) of nephrolithiasis for hemorrhagic stroke and ischemic stroke. In these analyses, crude (simple) and adjusted (for age, sex, income, region of residence, hypertension, diabetes, dyslipidemia, ischemic heart disease, and depression) models were used, and 95% confidence intervals (CIs) were calculated.

For the subgroup analyses, we divided the participants by age (20-39, 40-59, and 60+ years) and sex (men and women). In another subgroup analysis, we assessed the HRs of nephrolithiasis for hemorrhagic stroke and ischemic stroke within specific follow-up periods (≤ 1 year, 2-3 years, and > 3 years).

Two-tailed analyses were conducted, and P values less than 0.05 were regarded as indicative of significance. The results were statistically analyzed using SPSS v. 21.0 (IBM, Armonk, NY, USA).

## Results

The mean time from index date to hemorrhagic stroke was 71.8 months (SD = 41.4) in nephrolithiasis and 71.7 months (SD = 41.4) in control group. That of ischemic stroke was 69.8 months (SD = 41.4) in nephrolithiasis and 70.1 months (SD = 41.3) in control group. The rate of hemorrhagic stroke was not significantly higher in the nephrolithiasis group (0.8% [182/22,636]) than that in the control group (0.7% [678/90,544], P = 0.392), whereas the rate of ischemic stroke was significantly higher in the nephrolithiasis group (4.8% [1,090/21,546]) than that in the control group (4.3% [3,855/86,689], P < 0.001, Table 1). The two groups of participants were identical with respect to the general characteristics (age, sex, income, region of residence, hypertension, diabetes, and dyslipidemia histories) due to the matching procedure (P = 1.000). The rates of ischemic heart disease and a history of depression were higher in the nephrolithiasis group than those in the control group (P < 0.05 for each comparison).

The crude and adjusted HRs for hemorrhagic stroke were 1.07 (95% CI = 0.91-1.26, P = 0.395) and 1.07 (95% CI = 0.91-1.26, P = 0.427) in the nephrolithiasis group, respectively (Table 2). The crude and adjusted HRs for ischemic stroke were 1.14 (95% CI = 1.06-1.22) and 1.13 (95% CI = 1.06-1.21) in the nephrolithiasis group, respectively (P < 0.001 for each comparison).

In the subgroup analyses, none of the crude and adjusted HRs for hemorrhagic stroke reached statistical significance (Table 3). For ischemic stroke, the HRs of nephrolithiasis were significant for the young women and middle-aged men (P < 0.05 for each comparison). The adjusted HRs were 1.89 (95% CI = 1.04-3.47) in < 40-year-old women and 1.17 (95% CI = 1.04-1.33) in 40- to 59-year-old men in the nephrolithiasis group.

In another subgroup analysis, in the nephrolithiasis group, only the crude and adjusted HRs for ischemic stroke for a follow-up period of ≤ 1 year were statistically significant (adjusted HR = 1.30, 95% CI = 1.11-1.52, P = 0.001) (Table 4).

## Discussion

The present study demonstrated that nephrolithiasis increased the risk of ischemic stroke (adjusted HR = 1.13, 95% CI = 1.06-1.21). In the subgroup analyses by age and sex, this association was consistently observed only in the young women and middle-aged men. In another subgroup analysis, this association was significant for a follow-up period of ≤ 1 year after nephrolithiasis. No significant associations between nephrolithiasis and hemorrhagic stroke were observed.

The results of this study were similar to those of previous studies. Two prior population-based cohort studies reported increased HRs of stroke for nephrolithiasis patients (HR = 1.06, 95% CI = 1.01-1.11; HR = 1.43, 95% CI = 1.35-1.50).^11^,^16^ A cross-sectional study indicated that nephrolithiasis was associated with an odds ratio (OR) of 1.33 for stroke (95% CI = 1.01-1.74) 15. Two meta-analyses also indicated that nephrolithiasis patients had an increased risk of stroke (HR = 1.40, 95% CI = 1.20-1.64; relative risk = 1.21, 95% CI = 1.06-1.38).^17^,^18^

In the subgroup analyses, we found a relatively high HR in young women (adjusted HR = 1.89, 95% CI = 1.04-3.47) despite the smaller number of subjects in this group (n = 10,360) than that in other groups. Previously, an evident association between nephrolithiasis and stroke was identified in women.^11^,^18^ Although the association between nephrolithiasis and stroke in women has proven to be challenging to explain, the high prevalence of urinary tract infections in women could be a possible answer.^23^

This relationship could be derived from the effects of hypercalciuria, hyperoxaluria, and hypocitraturia.^10^ The common pathophysiology between vascular and renal calcification was suggested, because the vascular plaque had comparable constituent with renal Randall plaque, which is a stone nidus. 24 In addition, the shared pathophysiology of deficiencies in inhibitors of calcification in blood and urine of stroke and nephrolithiasis or chronic renal disease patients might contribute to this association.^26^,^27^

The association between nephrolithiasis and stroke could be explained based on common pathophysiologies. First, obesity and insulin resistance result in defective ammoniagenesis;^28^ therefore, diabetes could increase the risk of uric acid renal stones by inducing low urinary pH.^29^

Second, diabetes and hypertension could increase the risk of nephrolithiasis,^9^,^10^ which also increases the risk of stroke. Third, smoking could increase the risk of both nephrolithiasis and stroke.^30^,^31^

In this study, we found that nephrolithiasis was significantly associated with ischemic stroke but not hemorrhagic stroke. The possible mechanisms described above might act to promote ischemic stroke. Another potential explanation is higher statistical power for analyses of ischemic stroke due to the larger number of ischemic stroke events (n = 4,945) than hemorrhagic stroke events (n = 860). In this study, we found an association between nephrolithiasis and ischemic stroke during a follow-up period of ≤ 1 year, a finding that has not been reported in other studies.^11^,^16^ We did not observe this relationship for follow-up periods of 2-3 years and > 3 years, although our observations do not necessarily indicate that this relationship was only present shortly after nephrolithiasis.

The advantages of this study are consistent with those of our previous studies utilizing the HIRA-NSC.^32^-^34^ We used a very large, representative, nationwide population. Because NHIS data include all citizens of Korea, without exceptions, there were no participants lost during follow-up. The control group was randomly selected, with matching based on age, sex, income, region of residence, and prior medical history used to avoid confounding effects. An adjusted hazard model was used to further minimize the impact of confounders. We extended previous findings in that we divided stroke into hemorrhagic and ischemic stroke and analyzed risks of stroke by follow-up period.

This study has certain limitations. Despite the cohort study design, we could not exclude the effects of possible confounders that might have affected both nephrolithiasis and stroke. Because we do not have data regarding body mass index, smoking, and history of alcohol use, we could not adjust for these factors. Certain patients might not have visited a clinic for treatment of nephrolithiasis and/or stroke, and these patients might have been missed. Visits for nephrolithiasis might have increased the chance of stroke detection. Therefore, we performed an additional analysis for between > 3 months and 1 year after the detection of nephrolithiasis. The results of this analysis were consistent with our aforementioned findings (adjusted HR of ischemic stroke = 1.22, 95% CI = 1.01-1.48, P = 0.044, Supplementary Table S1).

## Conclusion

The nephrolithiasis patients had an elevated risk of ischemic stroke. In the subgroup analysis, this association was constant in young women and middle-aged men as well as during a follow-up period of ≤ 1 year. There was no significant association between nephrolithiasis and the risk of hemorrhagic stroke.

## Supplementary Material

Supplementary Table S1.

## Figures and Tables

**Table 1:** General Characteristics of Participants

Characteristics		Total participants		
		Nephrolithiasis (n, %)	Control (n, %)	P-value
Age (years old)				1.000
	20-24	847 (3.7)	3,388 (3.7)	
	25-29	1,603 (7.1)	6,412 (7.1)	
	30-34	2,317 (10.2)	9,268 (10.2)	
	35-39	2,746 (12.1)	10,984 (12.1)	
	40-44	2,964 (13.1)	11,856 (13.1)	
	45-49	3,067 (13.5)	12,268 (13.5)	
	50-54	2,801 (12.4)	11,204 (12.4)	
	55-59	2,226 (9.8)	8,904 (9.8)	
	60-64	1,732 (7.7)	6,928 (7.7)	
	65-69	1,183 (5.2)	4,732 (5.2)	
	70-74	675 (3.0)	2,700 (3.0)	
	75-79	309 (1.4)	1,236 (1.4)	
	80-84	127 (0.6)	508 (0.6)	
	85+	39 (0.2)	156 (0.2)	
Sex				1.000
	Male	14,670 (64.8)	58,680 (64.8)	
	Female	7,966 (35.2)	31,864 (35.2)	
Income				1.000
	1 (lowest)	253 (1.1)	1,012 (1.1)	
	2	1,341 (5.9)	5,364 (5.9)	
	3	1,427 (6.3)	5,708 (6.3)	
	4	1,582 (7.0)	6,328 (7.0)	
	5	1,646 (7.3)	6,584 (7.3)	
	6	1,929 (8.5)	7,712 (8.5)	
	7	2,286 (10.1)	9,144 (10.1)	
	8	2,570 (11.4)	10,280 (11.4)	
	9	2,865 (12.7)	11,460 (12.7)	
	10	3,174 (14.0)	12,696 (14.0)	
	11 (highest)	3,564 (15.7)	14,256 (15.7)	
Region of residence				1.000
	Urban	10,738 (47.4)	42,952 (47.4)	
	Rural	11,898 (52.6)	47,592 (52.6)	
Hypertension				1.000
	Yes	7,907 (34.9)	31,628 (34.9)	
	No	14,729 (65.1)	58,916 (65.1)	
Diabetes				1.000
	Yes	4,272 (18.9)	17,088 (18.9)	
	No	18,364 (81.1)	73,456 (81.1)	
Dyslipidemia				1.000
	Yes	6,576 (29.1)	26,304 (29.1)	
	No	16,060 (70.9)	64,240 (70.9)	
Ischemic heart disease				<0.001*
	Yes	1,356 (6.0)	4,578 (5.1)	
	No	21,280 (94.0)	85,966 (94.9)	
Depression				<0.001*
	Yes	1,922 (8.5)	6,685 (7.4)	
	No	20,714 (91.5)	83,859 (92.6)	
Hemorrhagic stroke				0.392
	Yes	182 (0.8)	678 (0.7)	
	No	22,454 (99.2)	89,866 (99.3)	
Ischemic stroke				<0.001*
	Yes	1,090 (4.8)	3,855 (4.3)	
	No	21,546 (95.2)	86,689 (95.7)	

*Chi-square test or Fisher's exact test. Significance at P < 0.05.

**Table 2:** Crude and adjusted hazard ratios (95% confidence interval) of nephrolithiasis for hemorrhagic stroke and ischemic stroke

Characteristics		Hemorrhagic stroke				Ischemic stroke			
		Crude	P-value	Adjusted†	P-value	Crude	P-value	Adjusted†	P-value
Nephrolithiasis			0.395		0.427		<0.001*		<0.001*
	Yes	1.07 (0.91-1.26)		1.07 (0.91-1.26)		1.14 (1.06-1.22)		1.13 (1.06-1.21)	
	No	1.00		1.00		1.00		1.00	

* Cox-proportional hazard regression model, Significance at P < 0.05. † Adjusted model for age, sex, income, region of residence, hypertension, diabetes, hyperlipidemia, ischemic heart disease, and depression histories.

**Table 3:** Subgroup analysis of crude and adjusted hazard ratios (95% confidence interval) of nephrolithiasis for hemorrhagic stroke and ischemic stroke

Characteristics		Hemorrhagic stroke				Ischemic stroke			
		Crude	P-value	Adjusted†	P-value	Crude	P-value	Adjusted†	P-value
**Young men (20-39 years old, n = 27,205)**									
Nephrolithiasis			0.640		0.642		0.811		0.787
	Yes	0.87 (0.48-1.58)		0.87 (0.48-1.58)		0.96 (0.68-1.36)		0.95 (0.67-1.35)	
	No	1.00		1.00		1.00		1.00	
**Young women (20-39 years old, n = 10,360)**									
Nephrolithiasis			0.217		0.200		0.023*		0.038*
	Yes	1.75 (0.72-4.25)		1.79 (0.74-4.35)		2.00 (1.10-3.65)		1.89 (1.04-3.47)	
	No	1.00		1.00		1.00		1.00	
**Middle aged men (40-59 years old, n = 35,060)**									
Nephrolithiasis			0.551		0.553		0.006*		0.012*
	Yes	0.91 (0.68-1.23)		0.91 (0.68-1.22)		1.19 (1.05-1.35)		1.17 (1.04-1.33)	
	No	1.00		1.00		1.00		1.00	
**Middle aged women (40-59 years old, n = 20,230)**									
Nephrolithiasis			0.157		0.181		0.049*		0.080
	Yes	1.32 (0.90-1.95)		1.30 (0.88-1.92)		1.19 (1.00-1.41)		1.17 (0.98-1.39)	
	No	1.00		1.00		1.00		1.00	
**Old men (60+ years old, n = 11,085)**									
Nephrolithiasis			0.443		0.449		0.248		0.288
	Yes	1.14 (0.81-1.61)		1.14 (0.81-1.61)		1.08 (0.95-1.23)		1.07 (0.94-1.22)	
	No	1.00		1.00		1.00		1.00	
**Old women (60+ years old, n = 9,240)**									
Nephrolithiasis			0.627		0.674		0.071		0.115
	Yes	1.10 (0.75-1.63)		1.09 (0.74-1.61)		1.14 (0.99-1.31)		1.12 (0.97-1.29)	
	No	1.00		1.00		1.00		1.00	

* Cox-proportional hazard regression model, Significance at P < 0.05 † Adjusted model for age, sex, income, region of residence, hypertension, diabetes, hyperlipidemia, ischemic heart disease, and depression histories

**Table 4:** Subgroup analysis of crude and adjusted hazard ratios (95% confidence interval) of nephrolithiasis for hemorrhagic stroke and ischemic stroke according to follow up periods

Characteristics		Hemorrhagic stroke				Ischemic stroke			
		Crude	P-value	Adjusted†	P-value	Crude	P-value	Adjusted†	P-value
**≤ 1 year**									
Nephrolithiasis			0.573		0.548		0.001*		0.001*
	Yes	0.89 (0.59-1.34)		0.88 (0.59-1.33)		1.31 (1.12-1.53)		1.30 (1.11-1.52)	
	No	1.00		1.00		1.00		1.00	
**2-3 year**									
Nephrolithiasis			0.215		0.215		0.120		0.145
	Yes	1.29 (0.86-1.94)		1.29 (0.86-1.94)		1.15 (0.96-1.38)		1.14 (0.96-1.37)	
	No	1.00		1.00		1.00		1.00	
**> 3 years**									
Nephrolithiasis			0.355		0.390		0.400		0.505
	Yes	1.11 (0.89-1.38)		1.10 (0.88-1.37)		1.04 (0.95-1.14)		1.03 (0.94-1.13)	
	No	1.00		1.00		1.00		1.00	

* Cox-proportional hazard regression model, Significance at P < 0.05. † Adjusted model for age, sex, income, region of residence, hypertension, diabetes, hyperlipidemia, ischemic heart disease, and depression historie.
