# Supplementary material for: Nephrolithiasis predicts ischemic stroke: A longitudinal follow-up study using a national sample cohort
Source: Int J Med Sci. 2019 Jul 21;16(8):1050–6. doi: 10.7150/ijms.34417 (PMC6743278; doi:10.7150/ijms.34417)
Supplement: Supplementary file 1 — Supplementary Table S1. [file ijmsv16p1050s1.pdf]

Supplementary Table S1 Crude and adjusted hazard ratios (95% confidence interval) of nephrolithiasis for hemorrhagic stroke and ischemic stroke in periods of 4 months through 12 months.

Characteristics	Hemorrhagic stroke				Ischemic stroke			
	Crude	P-value	Adjusted†	P-value	Crude	P-value	Adjusted†	P-value
Nephrolithiasis		0.522		0.503		0.035*		0.044*
Yes	0.85 (0.51-1.41)		0.84 (0.51-1.40)		1.23 (1.02-1.49)		1.22 (1.01-1.48)	
No	1.00		1.00		1.00		1.00	

* Cox-proportional hazard regression model, Significance at P < 0.05

† Adjusted model for age, sex, income, region of residence, hypertension, diabetes, hyperlipidemia, ischemic heart disease, and depression histories
